# Testosterone-related indices and adverse glucose-lipid metabolic status in men: a single-center retrospective study

**DOI:** 10.3389/fendo.2026.1930279

**Published:** 2026-08-28

**Authors:** Miaomiao Ma, Qi Zhang, Zulong Wang, Baojun Ju, Shiqi Wang, Zhang Hao, Kaibo Su, Rubing Chen, Wenbo Zhang

**Affiliations:** 1 Department of Andrology, The First Affiliated Hospital of Henan University of Chinese Medicine, Zhengzhou, China; 2 The First Clinical Medical College of Henan University of Chinese Medicine, Zhengzhou, China; 3 Department of Hematology and Oncology, The First Affiliated Hospital of Henan University of Chinese Medicine, Zhengzhou, China; 4 Department of Integrated Chinese and Western Medicine, Henan Cancer Hospital, Zhengzhou, China

**Keywords:** cross-sectional study, glucose metabolism, lipid metabolism, sex hormone-binding globulin, testosterone

## Abstract

**Background:**

Lower testosterone is often observed with abnormal glucose-lipid metabolism, but the clinical value of calculated testosterone fractions in metabolic assessment remains uncertain. This study evaluated cross-sectional associations of total testosterone (TT), calculated free testosterone (FT), free testosterone percentage (FT%), bioavailable testosterone (BioT), bioavailable testosterone percentage (BioT%), and sex hormone-binding globulin (SHBG) with glucose-lipid traits in male andrology outpatients.

**Methods:**

This single-center retrospective cross-sectional analysis included 420 adult male andrology outpatients with complete records for TT, SHBG, albumin, glucose, total cholesterol (TC), triglycerides (TG), high-density lipoprotein cholesterol (HDL-C), and low-density lipoprotein cholesterol (LDL-C). Age-adjusted partial Spearman correlations, rank-standardized regression, SHBG-adjusted models, restricted cubic spline analysis, age-stratified interaction analysis, sensitivity analyses, and exploratory machine learning-assisted validation were performed.

**Results:**

TT was inversely associated with glucose and TG and positively associated with HDL-C. FT% and BioT% were positively associated with TG and inversely associated with HDL-C, but these associations were markedly attenuated or reversed after additional adjustment for SHBG. Restricted cubic spline models supported approximately linear associations of TT with glucose, TG, and HDL-C. In machine learning-assisted validation, adverse glucose-lipid metabolic status was present in 285 participants (67.9%). The Age + TT + SHBG model and XGBoost showed modest discrimination, with AUCs of 0.691 and 0.688, respectively. SHAP analysis suggested SHBG and age were the strongest model contributors, but this ranking should be interpreted as exploratory because correlated predictors can affect SHAP-based importance estimates.

**Conclusions:**

In male andrology outpatients, lower TT was associated with an adverse glucose-lipid profile, particularly higher glucose and TG and lower HDL-C. Percentage-based calculated testosterone indices appeared to reflect SHBG-dependent redistribution of circulating testosterone rather than an independent favorable androgen state. Machine learning-assisted validation further supported the interpretive importance of SHBG, although prediction performance remained exploratory. These findings should be interpreted within a single-center outpatient cohort and should not be generalized directly to the broader male population.

## Introduction

Testosterone is a central indicator of male gonadal endocrine status, and variation in testosterone levels has been closely linked to the development and progression of abnormalities in glucose and lipid metabolism (1–3). Testosterone is not only the principal androgen required for male secondary sexual characteristics, sexual function, and reproductive endocrine homeostasis, but also an important metabolic regulator involved in body-fat distribution, insulin sensitivity, hepatic lipid synthesis, and skeletal-muscle glucose uptake (4, 5). In clinical practice, low testosterone is often accompanied by obesity, visceral adiposity, insulin resistance, dysglycemia, and dyslipidemia, all of which contribute to the pathophysiological basis of metabolic syndrome (MetS) and type 2 diabetes mellitus (T2DM) (6, 7). In andrology, testosterone assessment is commonly focused on erectile dysfunction, infertility, and late-onset hypogonadism (LOH) (8–10), whereas its potential cardiometabolic implications are often under-recognized. Clarifying the relationship between male testosterone status and glucose-lipid metabolism may therefore support more comprehensive health management and interdisciplinary risk intervention in men attending andrology clinics.

Previous studies of androgens and metabolism have mainly focused on the cross-sectional association between the absolute concentration of total testosterone (TT) and specific metabolic diseases (11, 12). In clinical laboratory reports, derived indices such as free testosterone (FT), free testosterone percentage (FT%), bioavailable testosterone (BioT), and bioavailable testosterone percentage (BioT%) are also frequently reported. These indices are usually calculated from fixed mathematical formulas based on TT, sex hormone-binding globulin (SHBG), and serum albumin (ALB), but whether they provide clinically useful information on glucose-lipid metabolism remains uncertain.

Against this background, we conducted a single-center retrospective cross-sectional study including 420 male outpatients. Using TT, FT, FT%, BioT, and BioT% as the principal testosterone-related variables, we first assessed their age-adjusted correlations with glucose, total cholesterol (TC), triglycerides (TG), high-density lipoprotein cholesterol (HDL-C), and low-density lipoprotein cholesterol (LDL-C). We then constructed SHBG-adjusted models to clarify the role of the transport protein in associations involving derived testosterone indices, and used restricted cubic spline (RCS) models to examine the non-linear geometry of associations between TT and glucose-lipid traits. Finally, age-stratified interaction analyses and multiple sensitivity analyses were performed to evaluate the robustness of these cross-sectional associations and to provide evidence for coordinated screening of testosterone status and metabolic risk in men.

## Materials and methods

### Study design and participants

This was a single-center retrospective cross-sectional analysis. The study population comprised male patients who attended the Andrology Outpatient Clinic of The First Affiliated Hospital of Henan University of Chinese Medicine between June 1, 2025 and June 1, 2026.

The inclusion criteria were as follows: (1) adult men aged 18 years or older; (2) complete laboratory records for TT, SHBG, and ALB; and (3) complete laboratory records for glucose, TC, TG, HDL-C, and LDL-C.

The exclusion criteria were as follows: (1) current use of exogenous androgen supplementation or use within the previous 3 months; (2) primary or secondary gonadal dysfunction, including congenital disorders of sexual development such as Klinefelter syndrome, pituitary tumors, hyperprolactinemia, bilateral cryptorchidism, impaired testosterone synthesis, or previous bilateral orchiectomy; (3) advanced malignant tumors; and (4) severe liver cirrhosis, acute or chronic liver failure, or nephrotic syndrome.

**Figure 1:**
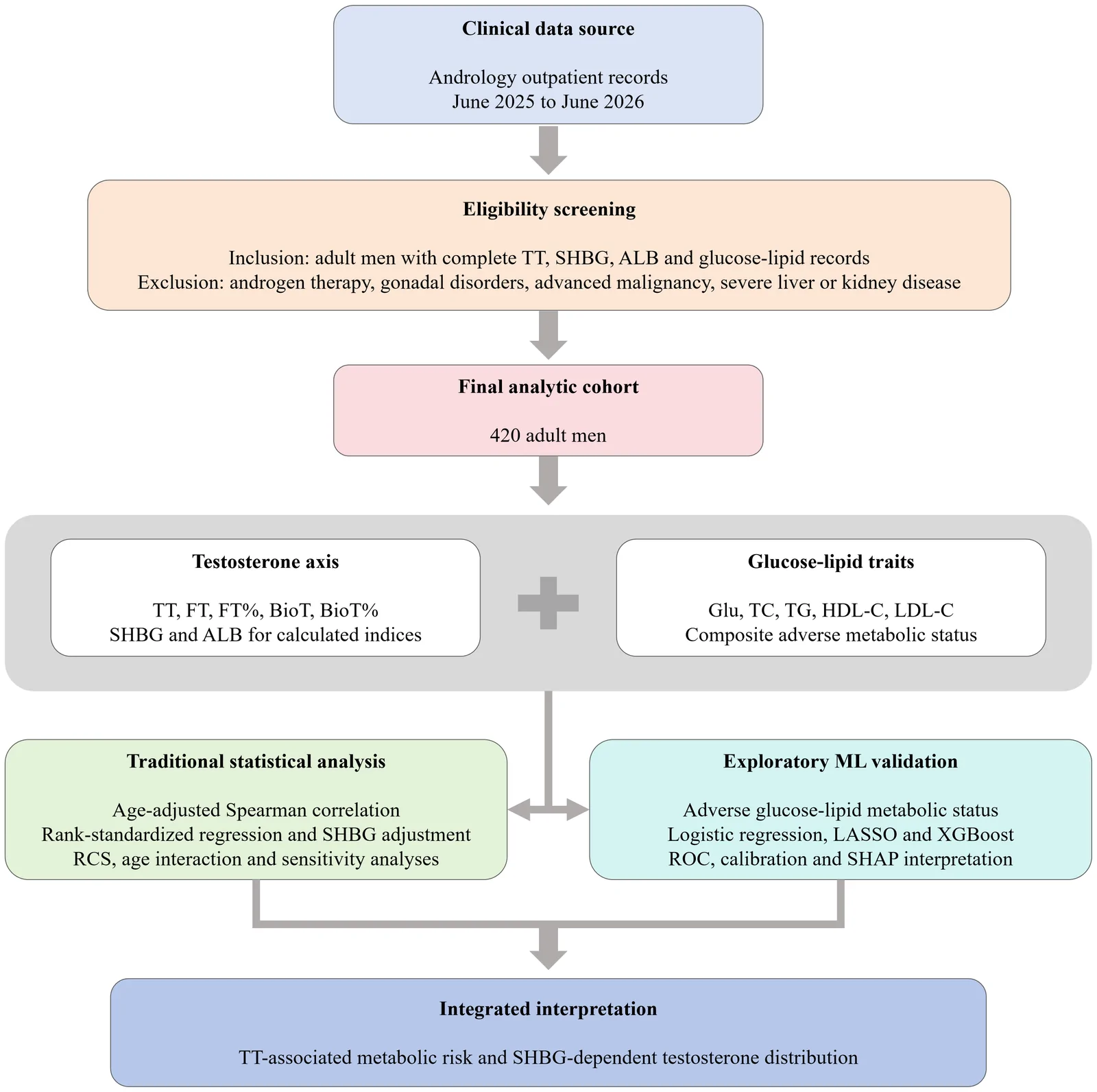
Study workflow and analytical framework.

Considering the clinical distribution of real-world data, extreme glucose-lipid values were not truncated at baseline, to preserve the representativeness of the sample. The potential influence of outliers on model estimates was assessed in subsequent sensitivity analyses by excluding the highest 1% of observations for selected metabolic traits. The study workflow is shown in **Figure 1**.

### Included variables

Testosterone-related indices included TT, FT, FT%, BioT, BioT%, and SHBG. FT, FT%, BioT, and BioT% were calculated from TT, SHBG, and ALB using the Vermeulen formula (13).

Glucose-lipid metabolic traits included glucose, TC, TG, HDL-C, and LDL-C.

### Statistical analysis

Continuous variables were described as the mean ± standard deviation, median (interquartile range), and range. Spearman rank correlation analysis was used to evaluate associations in a matrix restricted to five testosterone-related indices (TT, FT, FT%, BioT, and BioT%) and five glucose-lipid traits (glucose, TC, TG, HDL-C, and LDL-C).

To control for age, testosterone-related indices, glucose-lipid traits, and age were first rank-transformed, and partial Spearman correlation coefficients were then calculated using age-rank residuals. Rank-standardized multivariable linear regression models were fitted with each glucose-lipid trait as the dependent variable and each testosterone-related index as the independent variable, with age included as the first covariate. SHBG was subsequently added to the models to evaluate changes in the direction and magnitude of the regression coefficients. Multiple testing was controlled using the Benjamini-Hochberg false-discovery-rate procedure.

RCS models were fitted using natural cubic spline terms, and both overall and non-linear *P* values were reported. Age-stratified analyses were performed in three groups (<35 years, 35–44 years, and ≥45 years). Interaction was tested using models that included TT, age group, and the TT × age-group interaction term. Sensitivity analyses included exclusion of the highest 1% of glucose values, exclusion of the highest 1% of TG values, log-rank transformation models, and conventional *z*-score models. All tests were two-sided, and *P* < 0.05 was considered statistically significant. All analyses and figures were generated using R.

To evaluate whether testosterone-axis variables could identify adverse glucose-lipid metabolic status, an exploratory machine learning-assisted analysis was performed. Adverse glucose-lipid metabolic status was defined as glucose ≥ 6.1 mmol/L, TC ≥ 5.2 mmol/L, TG ≥ 1.7 mmol/L, HDL-C < 1.0 mmol/L, or LDL-C ≥ 3.4 mmol/L. Candidate predictors were restricted to age and testosterone-axis variables, including TT, FT, FT%, BioT, BioT%, and SHBG, to avoid leakage from the glucose-lipid traits used to define the outcome. The five component abnormalities and the number of abnormal components were also summarized to display heterogeneity within the composite endpoint. Logistic regression, LASSO logistic regression, and XGBoost models were internally validated using repeated five-fold cross-validation. Model discrimination was assessed using AUC, and calibration was evaluated by plotting observed outcome proportions against predicted probabilities. SHAP values were calculated from the final XGBoost model to interpret feature contributions.

## Results

### Baseline characteristics of the study population

A total of 420 participants were included in the final analysis. The mean age was 38.27 ± 8.77 years, and the median age was 36.00 years. The median concentrations of TT, FT, and BioT were 3.80 ng/mL, 0.09 ng/mL, and 2.23 ng/mL, respectively. Among glucose-lipid traits, the median values were 5.45 mmol/L for glucose, 5.06 mmol/L for TC, 1.38 mmol/L for TG, 1.26 mmol/L for HDL-C, and 3.12 mmol/L for LDL-C. Detailed baseline characteristics are shown in **Table 1**.

**Table 1:** Baseline characteristics of the analytic cohort (*n* = 420).

Variable	Unit	Mean ± SD	Median (IQR)	Range
Age	years	38.27 ± 8.77	36.00 (32.00-43.00)	20.00-72.00
TT	ng/mL	4.00 ± 1.42	3.80 (3.21-4.55)	0.41-19.06
Glucose	mmol/L	5.84 ± 1.70	5.45 (5.14-5.90)	4.26-17.41
TC	mmol/L	5.08 ± 1.03	5.06 (4.38-5.67)	2.56-9.67
TG	mmol/L	1.90 ± 1.98	1.38 (1.00-2.10)	0.32-19.59
HDL-C	mmol/L	1.28 ± 0.25	1.26 (1.09-1.44)	0.54-2.26
LDL-C	mmol/L	3.13 ± 0.73	3.12 (2.66-3.57)	1.18-6.11
SHBG	nmol/L	24.31 ± 10.53	22.85 (16.40-30.60)	6.33-67.80
FT	ng/mL	0.09 ± 0.05	0.09 (0.07-0.10)	0.01-0.80
FT%	%	2.33 ± 0.44	2.35 (2.05-2.63)	0.37-3.43
BioT	ng/mL	2.36 ± 1.14	2.23 (1.87-2.62)	0.15-20.30
BioT%	%	59.77 ± 10.91	60.10 (51.77-67.73)	30.30-91.50

TT, total testosterone; FT, free testosterone; BioT, bioavailable testosterone; SHBG, sex hormone-binding globulin; TC, total cholesterol; TG, triglycerides; HDL-C, high-density lipoprotein cholesterol; LDL-C, low-density lipoprotein cholesterol.

### Age-adjusted correlations between testosterone-related indices and glucose-lipid traits

Age-adjusted partial Spearman correlation analysis showed that TT was inversely correlated with glucose and TG and positively correlated with HDL-C. The correlation coefficients were -0.261 for TT with glucose, -0.259 for TT with TG, and 0.202 for TT with HDL-C, with all adjusted *P* values < 0.001. No significant correlations were observed between TT and either TC or LDL-C.

FT showed a weak inverse correlation with glucose (*rho* = -0.159, adjusted *P* = 0.00223), whereas its correlations with TG, HDL-C, TC, and LDL-C were not statistically significant. BioT was also inversely correlated with glucose (*rho* = -0.145, adjusted *P* = 0.00477), but its associations with the main lipid traits were weak.

**Figure 2:**
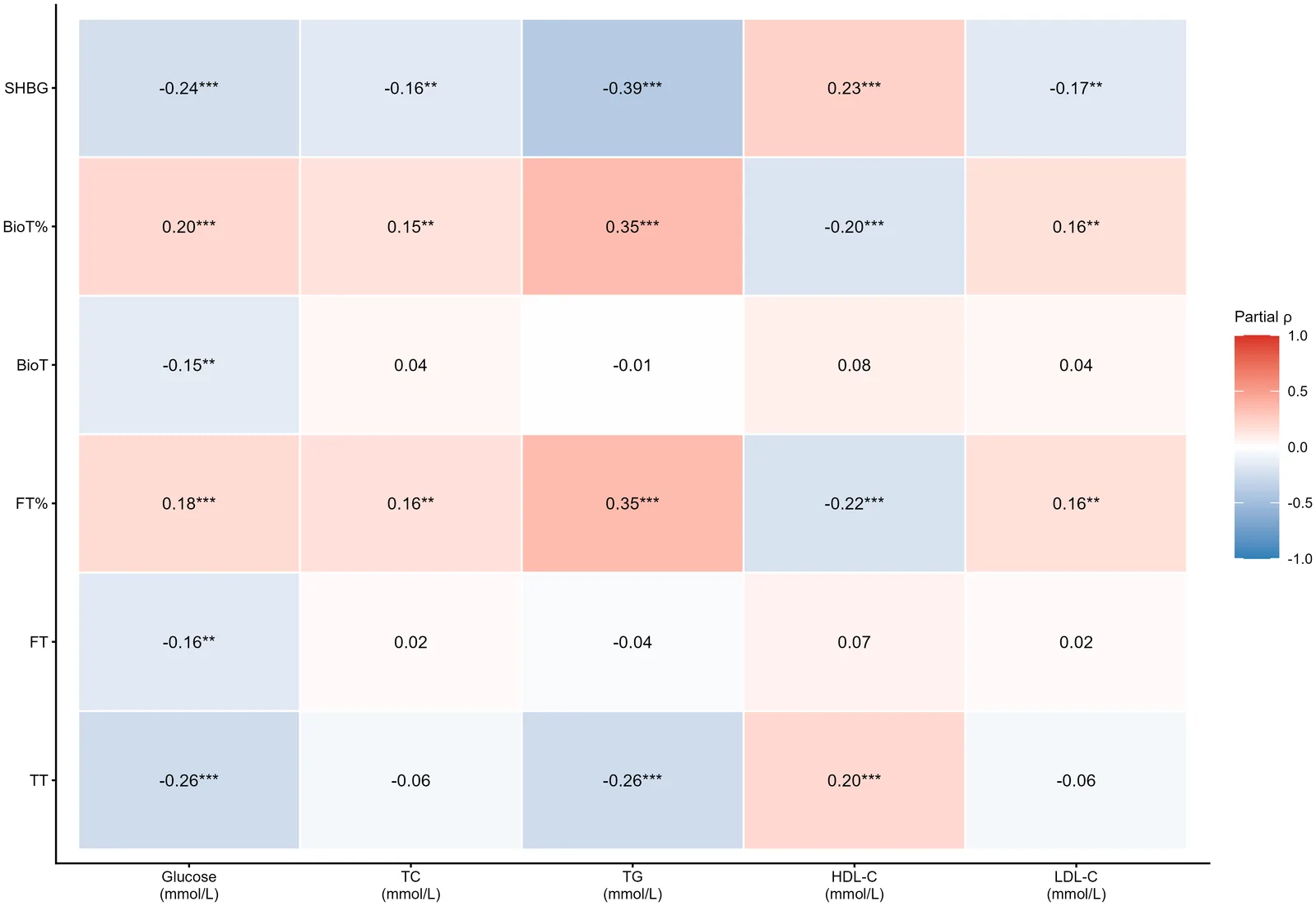
Age-adjusted partial Spearman correlations between testosterone-axis variables and metabolic traits. Colours indicate partial Spearman rho after adjustment for age rank residuals; red denotes positive associations and blue denotes inverse associations. Values inside cells are correlation coefficients. Asterisks indicate Benjamini-Hochberg-adjusted significance: *adjusted *p* < 0.05, **adjusted *p* < 0.01 and ***adjusted *p* < 0.001.

FT% and BioT% were positively correlated with TG and inversely correlated with HDL-C. The correlation coefficients of FT% and BioT% with TG were 0.350 and 0.348, respectively, with both adjusted *P* < 0.001. The corresponding coefficients with HDL-C were -0.216 and -0.200, respectively, with both adjusted *P* < 0.001. These results are shown in **Table 2** and **Figure 2**.

**Table 2:** Age-adjusted partial Spearman correlations between testosterone-axis variables and metabolic traits (*n* = 420).

Hormone	Glucose (*P* value)	TC (*P* value)	TG (*P* value)	HDL-C (*P* value)	LDL-C (*P* value)
TT	-0.261 (< 0.001)	-0.062 (0.27311)	-0.259 (< 0.001)	0.202 (< 0.001)	-0.062 (0.27311)
FT	-0.159 (0.00223)	0.025 (0.64825)	-0.035 (0.53316)	0.068 (0.23931)	0.024 (0.64825)
FT%	0.183 (< 0.001)	0.160 (0.00223)	0.350 (< 0.001)	-0.216 (< 0.001)	0.162 (0.00222)
BioT	-0.145 (0.00477)	0.039 (0.50283)	-0.012 (0.80760)	0.079 (0.16799)	0.042 (0.48337)
BioT%	0.200 (< 0.001)	0.152 (0.00321)	0.348 (< 0.001)	-0.200 (< 0.001)	0.157 (0.00232)

Cells show partial Spearman rho with the Benjamini-Hochberg-adjusted *P* value in parentheses. Partial Spearman *rho* was calculated after rank transformation and adjustment for age rank residuals. Pairwise complete observations were used for each comparison.

### Age-adjusted multivariable regression analysis

The age-adjusted rank-standardized regression results were broadly consistent with the partial Spearman correlation analysis. TT was inversely associated with glucose and TG and positively associated with HDL-C. The standardized *β* coefficients for TT were -0.257 for glucose, -0.258 for TG, and 0.202 for HDL-C, with all adjusted *P* < 0.001.

**Figure 3:**
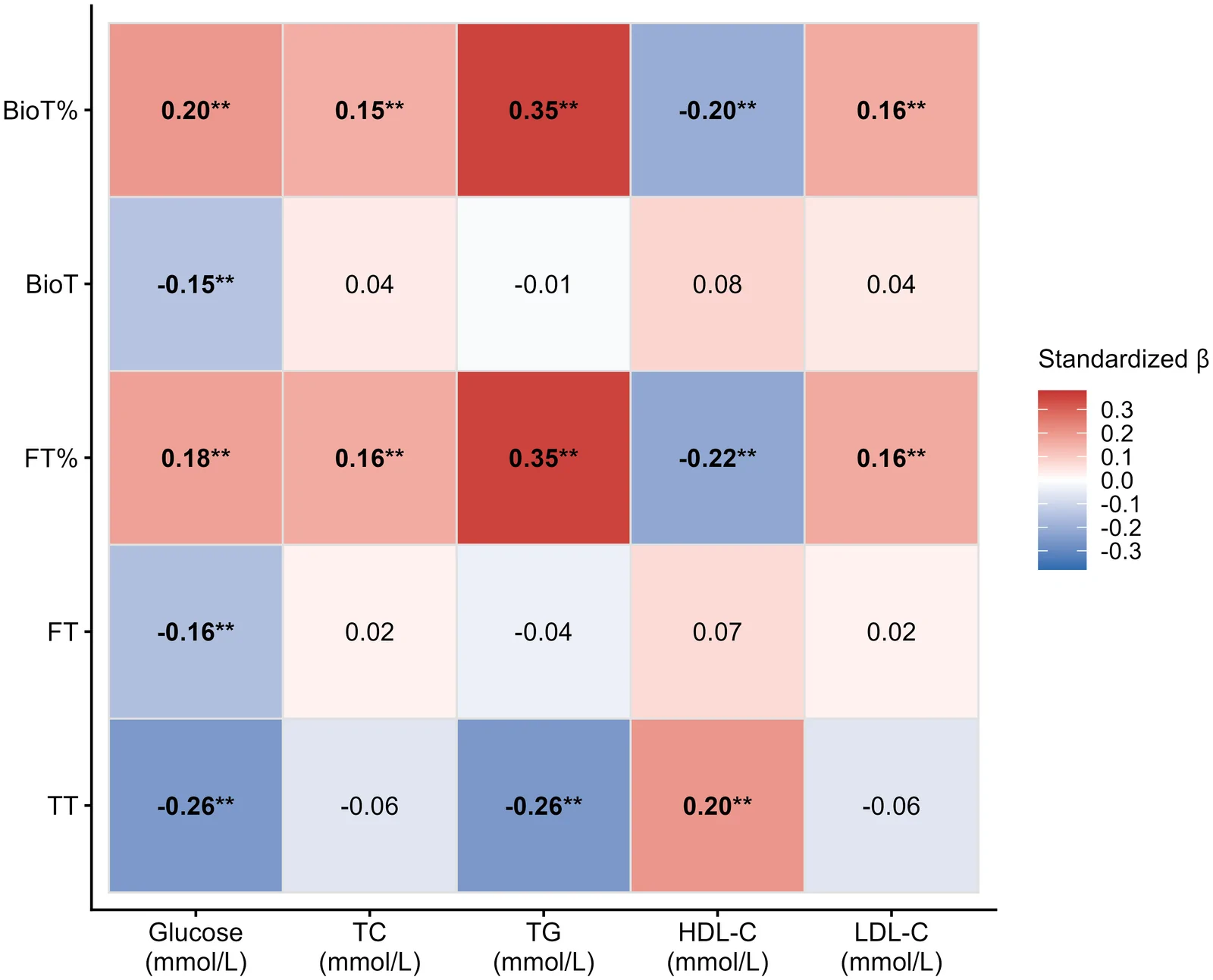
Heatmap of age-adjusted multivariable regression coefficients for testosterone-related indices and metabolic traits. Cells show standardized β coefficients from age-adjusted multivariable regression models using each metabolic trait as the dependent variable and each testosterone-related index as the independent variable. Red indicates positive associations and blue indicates inverse associations. Asterisks indicate Benjamini-Hochberg-adjusted significance: *q < 0.05, **q < 0.01, and ***q < 0.001. TT, total testosterone; FT, free testosterone; BioT, bioavailable testosterone; TC, total cholesterol; TG, triglycerides; HDL-C, high-density lipoprotein cholesterol; LDL-C, low-density lipoprotein cholesterol.

FT% and BioT% were positively associated with TG and inversely associated with HDL-C. The standardized β coefficients of both FT% and BioT% for TG were 0.354, with both adjusted *P* < 0.001. The standardized β coefficients of FT% and BioT% for HDL-C were -0.218 and -0.204, respectively, with both adjusted *P* < 0.001. The age-adjusted multivariable regression models are presented in **Table 3** and **Figure 3**.

**Table 3:** Age-adjusted multivariable regression models for testosterone-related indices and metabolic traits (*n* = 420).

Predictor	Metabolic trait (mmol/L)	Standardized *β*	Unstandardized *β* (95% CI)	*P* value	BH-adjusted *P* value	Adjusted *R^2^*
TT	Glucose	-0.257	-0.176 (-0.290 to -0.061)	< 0.001	< 0.001	0.109
FT	Glucose	-0.158	-1.803 (-5.432 to 1.826)	0.00109	0.00226	0.067
FT%	Glucose	0.181	0.593 (0.215 to 0.971)	< 0.001	< 0.001	0.075
BioT	Glucose	-0.146	-0.076 (-0.220 to 0.068)	0.0029	0.00483	0.063
BioT%	Glucose	0.2	0.024 (0.008 to 0.039)	< 0.001	< 0.001	0.081
TT	TC	-0.062	-0.048 (-0.118 to 0.022)	0.20701	0.27382	0.009
FT	TC	0.025	-0.081 (-2.282 to 2.121)	0.6099	0.64868	0.005
FT%	TC	0.161	0.292 (0.062 to 0.522)	0.00105	0.00226	0.03
BioT	TC	0.04	-0.002 (-0.090 to 0.085)	0.42293	0.50349	0.006
BioT%	TC	0.155	0.012 (0.003 to 0.021)	0.00182	0.00325	0.028
TT	TG	-0.258	-0.242 (-0.375 to -0.109)	< 0.001	< 0.001	0.075
FT	TG	-0.036	-2.296 (-6.528 to 1.936)	0.46971	0.53377	0.01
FT%	TG	0.354	1.188 (0.757 to 1.619)	< 0.001	< 0.001	0.13
BioT	TG	-0.012	-0.083 (-0.252 to 0.085)	0.80783	0.80783	0.009
BioT%	TG	0.354	0.051 (0.034 to 0.068)	< 0.001	< 0.001	0.129
TT	HDL-C	0.202	0.027 (0.010 to 0.044)	< 0.001	< 0.001	0.047
FT	HDL-C	0.069	-0.018 (-0.561 to 0.524)	0.16324	0.24006	0.012
FT%	HDL-C	-0.218	-0.129 (-0.185 to -0.073)	< 0.001	< 0.001	0.053
BioT	HDL-C	0.08	-0.000 (-0.022 to 0.021)	0.10793	0.16865	0.013
BioT%	HDL-C	-0.204	-0.005 (-0.007 to -0.003)	< 0.001	< 0.001	0.047
TT	LDL-C	-0.062	-0.033 (-0.083 to 0.016)	0.20811	0.27382	0.002
FT	LDL-C	0.025	0.081 (-1.480 to 1.642)	0.62273	0.64868	-0.001
FT%	LDL-C	0.164	0.195 (0.031 to 0.358)	< 0.001	0.00225	0.024
BioT	LDL-C	0.043	0.005 (-0.057 to 0.067)	0.38726	0.48408	0
BioT%	LDL-C	0.161	0.008 (0.002 to 0.015)	0.00123	0.00236	0.023

Rank-standardized metabolic traits were modeled as dependent variables and rank-standardized testosterone-related indices as predictors, with age included as a covariate. Standardized *β* coefficients represent rank-based associations. Unstandardized β coefficients and 95% CIs are provided in the revised table, and *P* values were two-sided and adjusted using the Benjamini-Hochberg method.

### Trends across TT and SHBG quartiles

**Figure 4:**
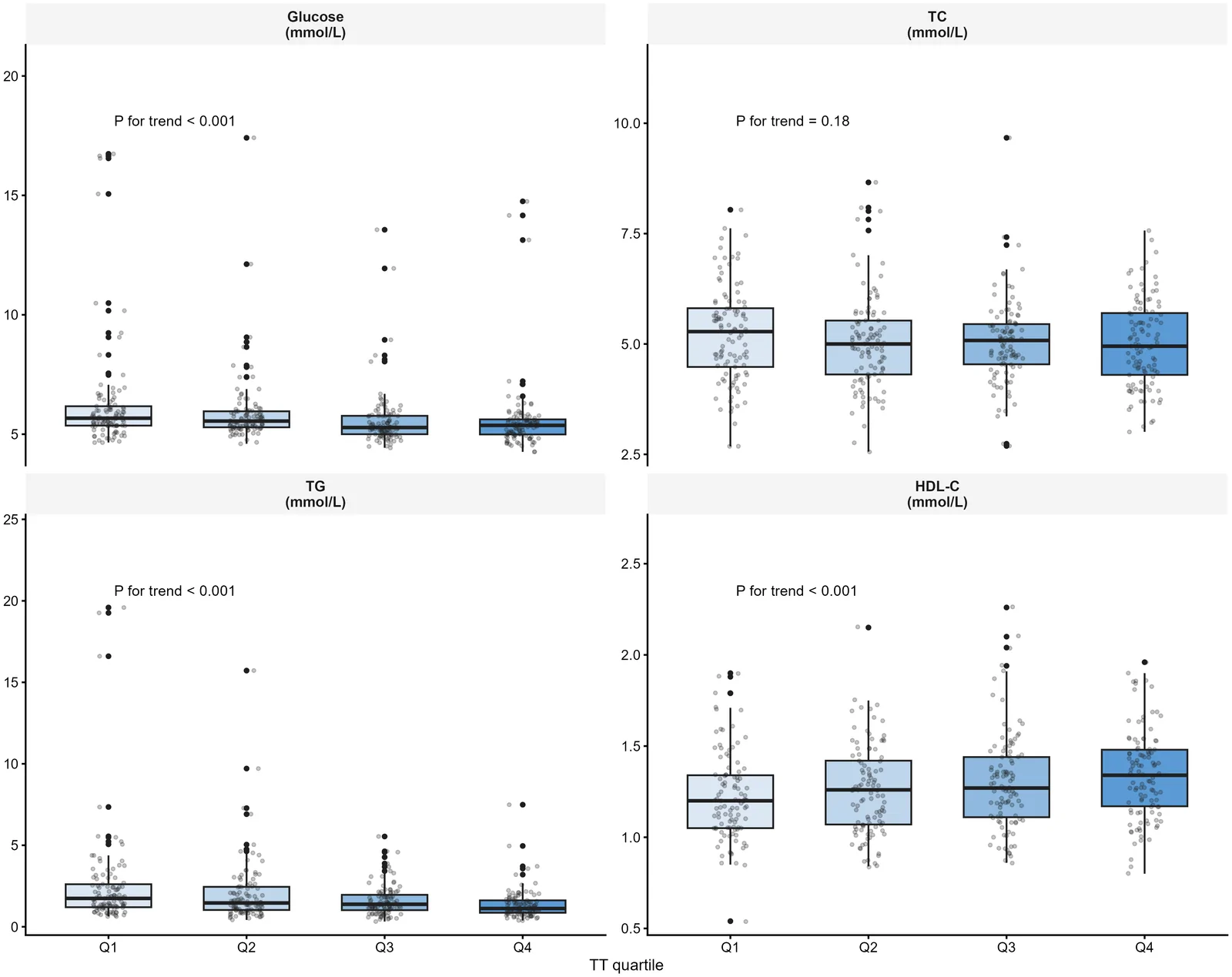
Metabolic profiles across TT quartiles.Box plots show the distributions of Glucose, TC, TG and HDL-C across TT quartiles; boxes indicate the median and interquartile range, whiskers extend to 1.5 times the interquartile range and points show individual observations. *P* for trend was calculated using Spearman correlation with quartile order as an ordinal variable.

When participants were grouped by TT quartiles, glucose and TG decreased across increasing TT quartiles, whereas HDL-C increased. No clear linear trend was observed for TC. These patterns were consistent with the correlation results showing inverse associations of TT with glucose and TG and a positive association with HDL-C. The distributions of major metabolic traits across TT quartiles are shown in **Figure 4**.

**Figure 5:**
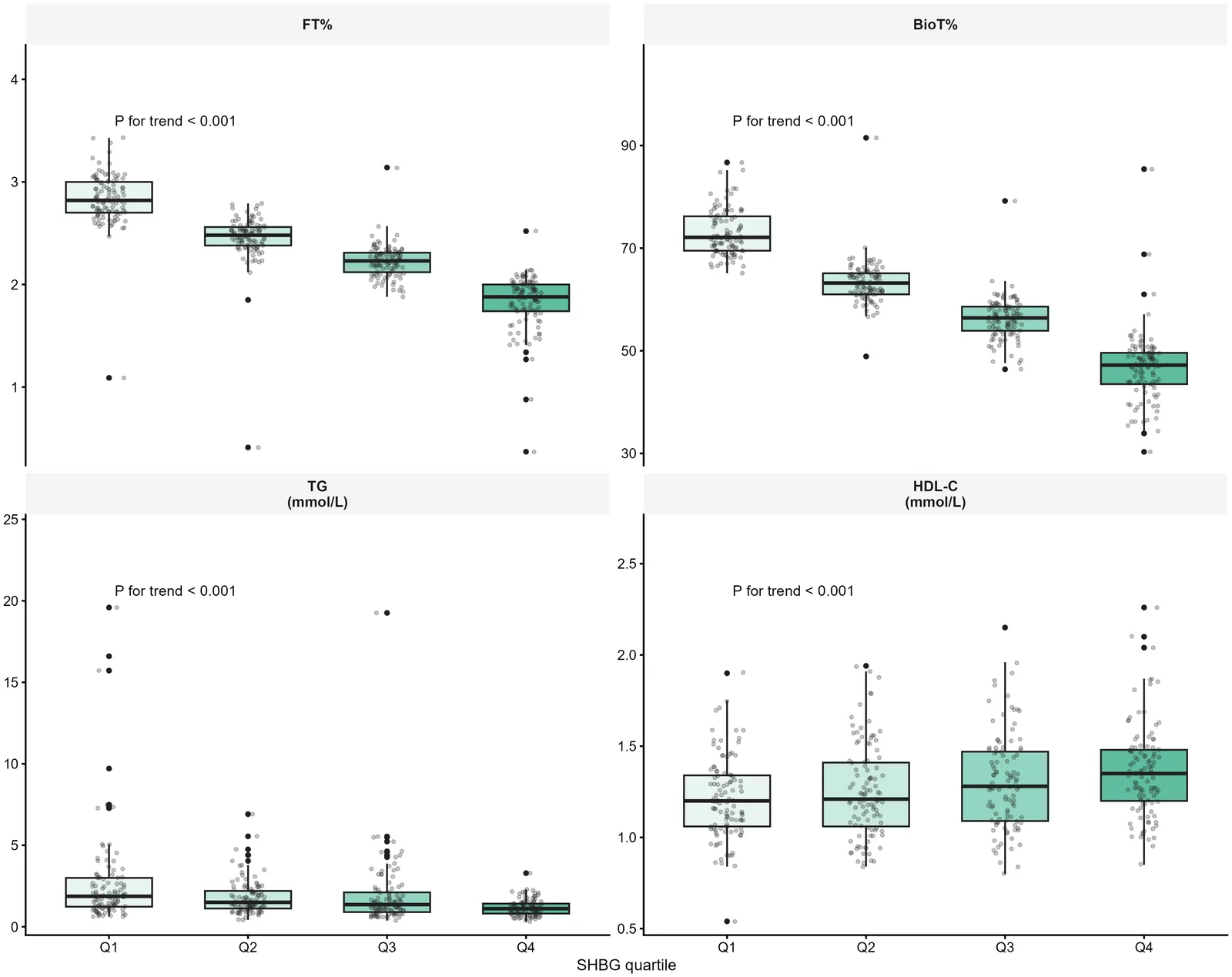
Testosterone fractions and lipid traits across SHBG quartiles.Box plots show FT%, BioT%, TG and HDL-C across SHBG quartiles; boxes indicate the median and interquartile range, whiskers extend to 1.5 times the interquartile range and points show individual observations. *P* for trend was calculated using Spearman correlation with quartile order as an ordinal variable.

When participants were grouped by SHBG quartiles, FT% and BioT% decreased across increasing SHBG quartiles. TG also decreased across SHBG quartiles, whereas HDL-C increased. These findings suggest that the associations of FT% and BioT% with TG and HDL-C may be related to the influence of SHBG on testosterone distribution. Trends in FT%, BioT%, TG, and HDL-C across SHBG strata are shown in **Figure 5**.

### Interpretation of SHBG-adjusted models

To further evaluate the role of SHBG in these associations, SHBG was added to the age-adjusted models. After SHBG adjustment, the association between TT and TG was markedly attenuated, with the standardized *β* changing from -0.258 to -0.054 and losing statistical significance. The association between TT and HDL-C was also attenuated, with the standardized *β* changing from 0.202 to 0.103; the *P* value after SHBG adjustment was 0.076.

For FT% and BioT%, the associations with TG and HDL-C also changed after SHBG was included. For FT%, the age-adjusted *β* for TG was 0.354, but changed to -0.135 after SHBG adjustment and was no longer significant. For BioT%, the age-adjusted *β* for TG was 0.354, but changed to -0.220 after SHBG adjustment and was not significant. The age-adjusted *β* of FT% for HDL-C was -0.218, but changed to 0.017 after SHBG adjustment. The age-adjusted *β* of BioT% for HDL-C was -0.204, but changed to 0.204 after SHBG adjustment and was not statistically significant. The SHBG-adjusted model results are shown in **Table 4**.

**Table 4:** SHBG-adjusted mechanistic models for testosterone-related indices and metabolic traits (*n* = 420).

Predictor	Metabolic trait	Age-adjusted *β*	Age-adjusted *P* value	SHBG-adjusted *β*	SHBG-adjusted *P* value	SHBG-adjusted *q* value	Absolute *β* change after SHBG (%)	Direction after SHBG adjustment	SHBG *β* in same model	SHBG *P* value in same model
TT	Glucose	-0.257	< 0.001	-0.184	0.00113	0.00938	-28.2	↓	-0.129	0.02427
FT	Glucose	-0.158	0.00109	-0.171	< 0.001	0.00704	8.1	↑	-0.244	< 0.001
FT%	Glucose	0.181	< 0.001	-0.334	0.01519	0.09491	84.4	**↔**	-0.549	< 0.001
BioT	Glucose	-0.146	0.0029	-0.164	< 0.001	0.00706	12.7	↑	-0.247	< 0.001
BioT%	Glucose	0.2	< 0.001	-0.247	0.10173	0.31792	23.8	**↔**	-0.468	0.00191
TT	TC	-0.062	0.20701	0.045	0.44964	0.70256	-27.9	**↔**	-0.189	0.00163
FT	TC	0.025	0.6099	0.017	0.7329	0.85238	-33.8	↓	-0.163	0.00102
FT%	TC	0.161	0.00105	0.07	0.62469	0.82197	-56.7	↓	-0.098	0.49556
BioT	TC	0.04	0.42293	0.028	0.5721	0.79458	-30.1	↓	-0.161	0.00112
BioT%	TC	0.155	0.00182	-0.012	0.94062	0.97411	-92.5	**↔**	-0.174	0.26344
TT	TG	-0.258	< 0.001	-0.054	0.32161	0.61847	-78.9	↓	-0.363	< 0.001
FT	TG	-0.036	0.46971	-0.057	0.2146	0.48772	58.4	↑	-0.397	< 0.001
FT%	TG	0.354	< 0.001	-0.135	0.31144	0.61847	-61.9	**↔**	-0.521	< 0.001
BioT	TG	-0.012	0.80783	-0.042	0.3643	0.65054	244.7	↑	-0.397	< 0.001
BioT%	TG	0.354	< 0.001	-0.22	0.13188	0.36635	-38	**↔**	-0.602	< 0.001
TT	HDL-C	0.202	< 0.001	0.103	0.07601	0.3167	-49	↓	0.176	0.00287
FT	HDL-C	0.069	0.16324	0.082	0.09075	0.31792	18.2	↑	0.239	< 0.001
FT%	HDL-C	-0.218	< 0.001	0.017	0.90563	0.97411	-92.4	**↔**	0.251	0.07607
BioT	HDL-C	0.08	0.10793	0.099	0.0434	0.21702	22.6	↑	0.242	< 0.001
BioT%	HDL-C	-0.204	< 0.001	0.204	0.18582	0.46455	0	**↔**	0.427	0.00547
TT	LDL-C	-0.062	0.20811	0.048	0.41252	0.68754	-21.7	**↔**	-0.196	0.00113
FT	LDL-C	0.025	0.62273	0.016	0.7501	0.85238	-36	↓	-0.167	< 0.001
FT%	LDL-C	0.164	< 0.001	0.054	0.704	0.85238	-66.8	↓	-0.117	0.41567
BioT	LDL-C	0.043	0.38726	0.031	0.53316	0.78406	-28.6	↓	-0.166	< 0.001
BioT%	LDL-C	0.161	0.00123	0.005	0.97411	0.97411	-96.8	↓	-0.163	0.29625

The absolute *β* change was calculated as 100 x (absolute *β* after SHBG adjustment-absolute *β* before SHBG adjustment)/absolute *β* before SHBG adjustment. Direction after SHBG adjustment was classified as: ↑strengthened, ↓attenuated, **↔**reversed.

### RCS, age-stratified interaction, and sensitivity analyses

**Figure 6:**
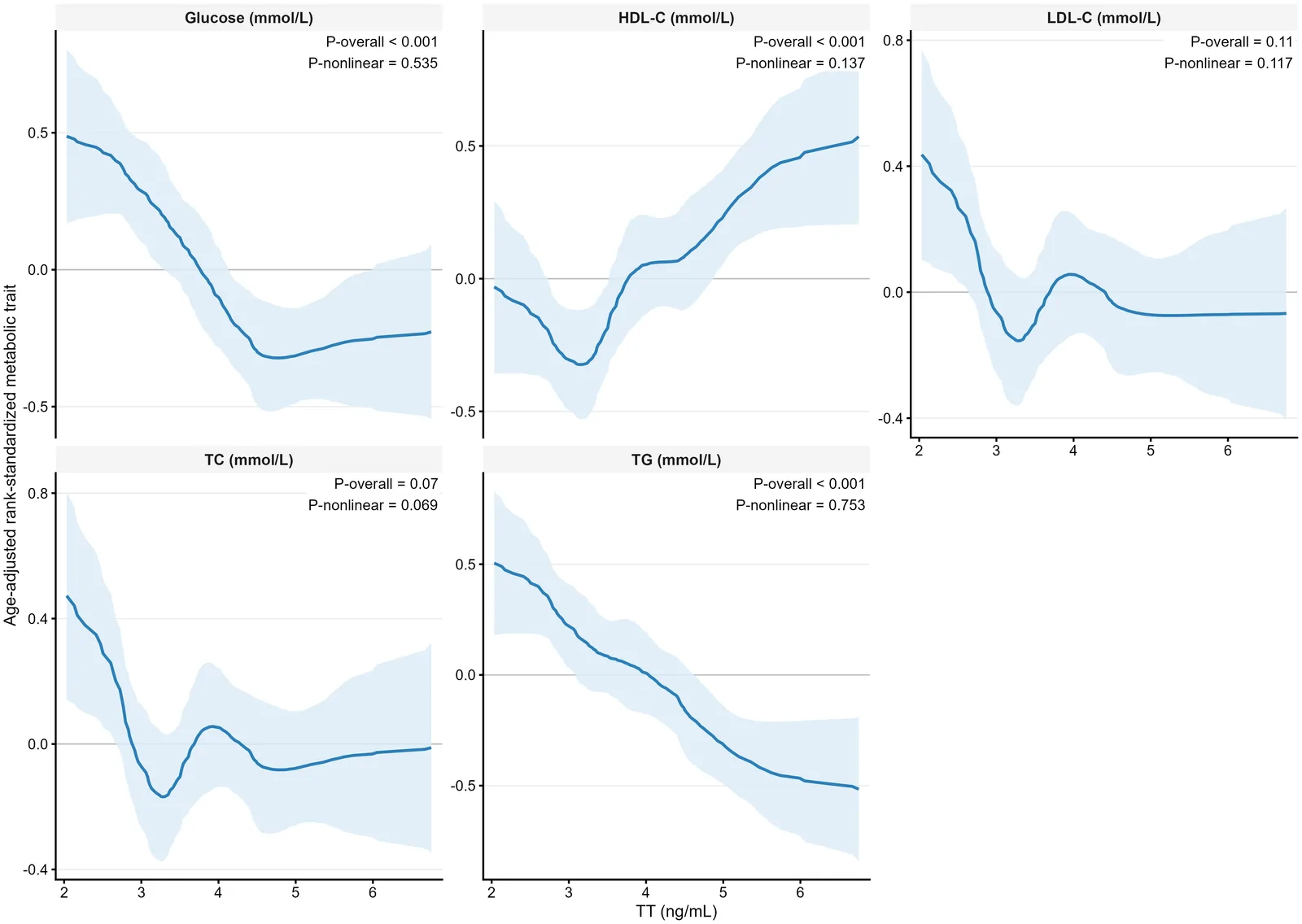
Restricted cubic spline analysis of TT and glucose-lipid traits. Curves show the age-adjusted association between TT and rank-standardized metabolic traits using natural cubic spline terms. Shaded areas indicate 95% confidence intervals. P-overall tests the overall association of the spline terms, and P-nonlinear compares the spline model with the linear model.

To further assess whether non-linear associations existed between TT and glucose-lipid traits, RCS analysis was performed. TT showed significant overall associations with glucose, TG, and HDL-C (*P*-overall < 0.001), whereas its overall associations with TC and LDL-C were not statistically significant. In tests for non-linearity, *P*-nonlinear values for glucose, TG, HDL-C, and LDL-C were > 0.05. Although the spline curve for TC showed slight local curvature, the present data do not support a biologically meaningful non-linear association between TT and TC. Age-adjusted spline curves for associations between TT and glucose-lipid traits are shown in **Figure 6**.

Age-stratified analysis showed that the directions of the associations of TT with glucose, TG, and HDL-C were generally consistent across age groups. The association between TT and glucose was more apparent in the <35-year and 35-44-year groups, but did not reach statistical significance in the ≥45-year group. TT was inversely associated with TG in all three age groups. TT was significantly and positively associated with HDL-C in the <35-year group, and the direction was consistent in the 35-44-year and ≥45-year groups, although statistical significance was not reached in those strata. *P* values for interaction were > 0.05 for all glucose-lipid traits, indicating no clear evidence of age interaction. Age-stratified and interaction analyses for TT and glucose-lipid traits are shown in **Table 5**.

**Table 5:** Age-stratified and interaction analysis for TT (*n* = 420).

Metabolic trait	<35 years *β* (*P* value)	35–44 years *β* (*P* value)	≥45 years *β* (*P* value)	*P* for interaction
Glucose	-0.346 (< 0.001)	-0.217 (0.00374)	-0.140 (0.18790)	0.239
HDL-C	0.274 (< 0.001)	0.138 (0.06036)	0.172 (0.10674)	0.598
LDL-C	-0.046 (0.56920)	-0.114 (0.13086)	-0.029 (0.78661)	0.688
TC	-0.012 (0.88757)	-0.161 (0.03207)	-0.006 (0.95283)	0.278
TG	-0.298 (< 0.001)	-0.268 (< 0.001)	-0.214 (0.04347)	0.892

Cells show *β* coefficients and *P* values from rank-standardized regression models within each age stratum. *β* represents the age-adjusted rank-standardized regression coefficient for TT. *P* for interaction was obtained from models including TT, age group and the TT × age-group interaction term.

**Figure 7:**
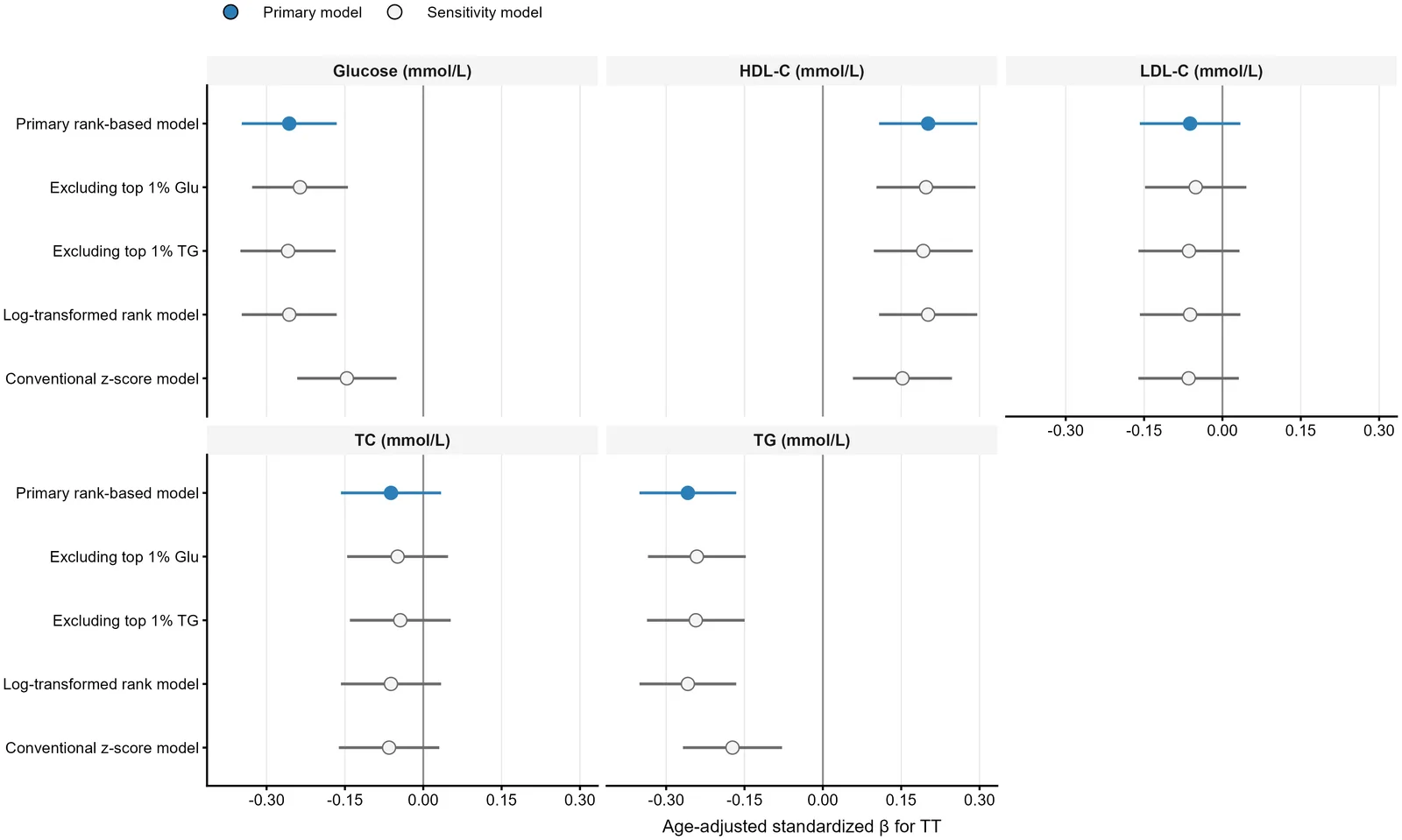
Sensitivity analysis of TT associations with glucose-lipid traits. Points indicate age-adjusted standardized β coefficients for TT, and horizontal lines indicate 95% confidence intervals. The primary rank-based model was compared with models excluding the top 1% of Glu or TG values and models using log-rank or conventional z-score transformations.

Sensitivity analyses showed that the direction and statistical significance of the associations of TT with glucose, TG, and HDL-C were generally stable after excluding the highest 1% of glucose values or the highest 1% of TG values. Results from log-rank transformation models were consistent with the main model for glucose, TG, and HDL-C. In conventional z-score models, the magnitude of the associations was attenuated, but TT remained significantly associated with glucose, TG, and HDL-C. TT did not show stable significant associations with TC or LDL-C across sensitivity models. The sensitivity analysis is shown in **Figure 7**.

### Machine learning-assisted validation of adverse glucose-lipid metabolic status

Adverse glucose-lipid metabolic status was present in 285 of 420 participants (67.9%). Elevated TC, elevated TG, elevated LDL-C, elevated glucose, and low HDL-C were observed in 182 (43.3%), 155 (36.9%), 148 (35.2%), 74 (17.6%), and 44 (10.5%) participants, respectively. The number of abnormal components was 0 in 135 participants (32.1%), 1 in 89 (21.2%), 2 in 100 (23.8%), 3 in 73 (17.4%), 4 in 20 (4.8%), and 5 in 3 (0.7%), indicating substantial heterogeneity within the composite endpoint. The outcome definition and model performance are shown in **Table 6**.

**Table 6:** Internal validation performance of traditional and machine learning models for adverse glucose-lipid metabolic status.

Model	Predictors	AUC (95% CI)	Sensitivity	Specificity	Accuracy	Brier score
Age only	Age	0.569 (0.508-0.629)	0.737	0.43	0.638	0.217
Age + TT	Age, TT	0.606 (0.548-0.664)	0.747	0.474	0.66	0.213
Age + TT + SHBG	Age, TT, SHBG	0.691 (0.634-0.747)	0.796	0.57	0.724	0.197
LASSO	Age, TT, FT, FT%, BioT, BioT%, SHBG	0.677 (0.621-0.734)	0.782	0.548	0.707	0.21
XG Boost	Age, TT, FT, FT%, BioT, BioT%, SHBG	0.688 (0.634-0.743)	0.751	0.578	0.695	0.226

Adverse glucose-lipid metabolic status was defined as glucose ≥ 6.1 mmol/L, TC ≥ 5.2 mmol/L, TG ≥ 1.7 mmol/L, HDL-C < 1.0 mmol/L, LDL-C ≥ 3.4 mmol/L, or any combination of these abnormalities. Models were internally validated using repeated five-fold cross-validation. AUC, area under the receiver operating characteristic curve; BioT, bioavailable testosterone; FT, free testosterone; SHBG, sex hormone-binding globulin; TT, total testosterone.

**Figure 8:**
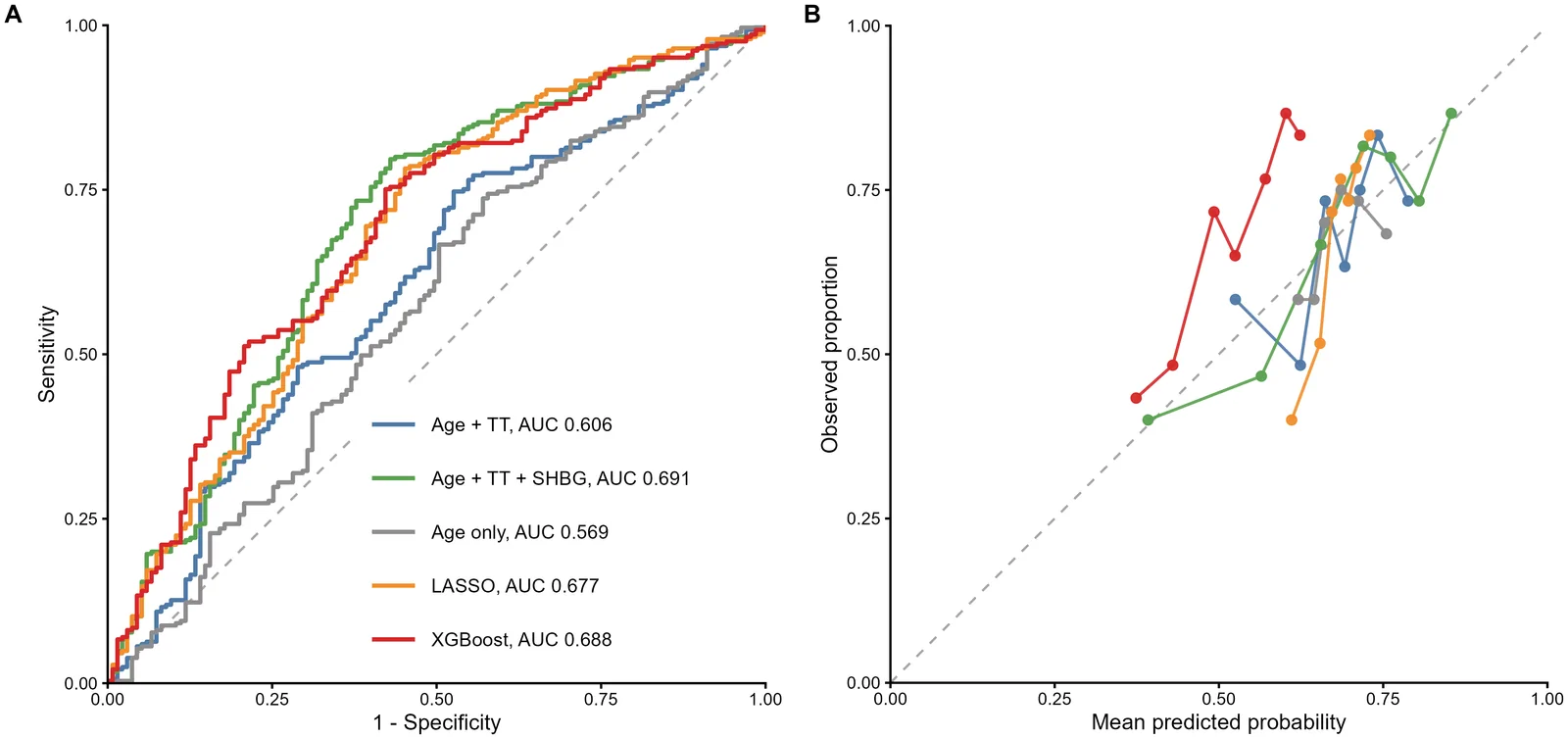
Internal validation of testosterone-axis models for adverse glucose-lipid metabolic status. Receiver operating characteristic curves **(A)** and calibration plots **(B)** show the performance of traditional regression and machine learning models for identifying adverse glucose-lipid metabolic status. Predictions were obtained from repeated five-fold cross-validation. The outcome was defined as the presence of elevated glucose, elevated TC, elevated TG, low HDL-C, or elevated LDL-C according to predefined clinical thresholds.

In repeated five-fold cross-validation, testosterone-axis models showed modest discrimination for adverse glucose-lipid metabolic status. The AUCs were 0.606 (95% CI, 0.548-0.664) for Age + TT, 0.691 (95% CI, 0.634-0.747) for Age + TT + SHBG, 0.677 (95% CI, 0.621-0.734) for LASSO, and 0.688 (95% CI, 0.634-0.743) for XGBoost. The ROC and calibration results are shown in **Figure 8**.

**Figure 9:**
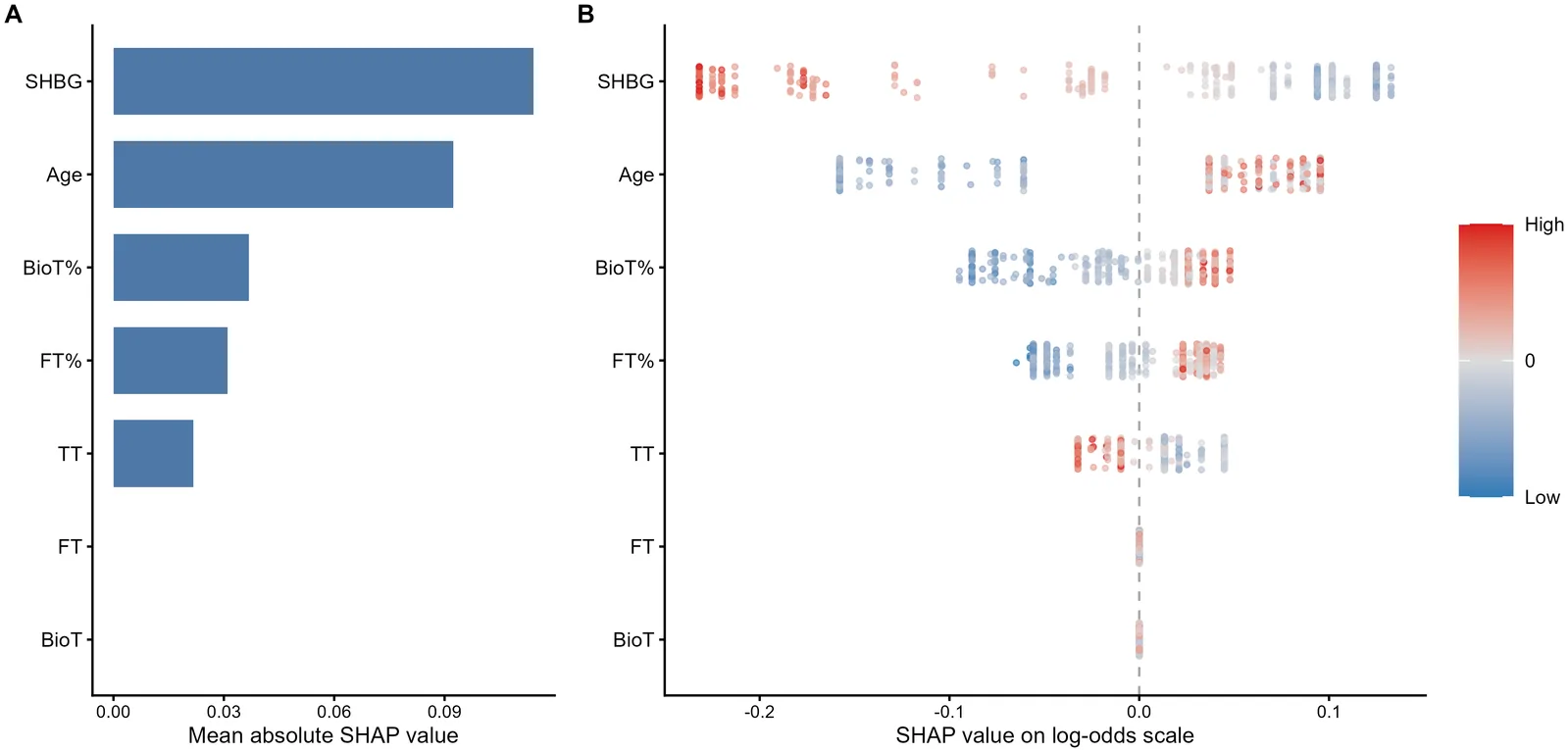
SHAP-based interpretation of the XGBoost model. Mean absolute SHAP values **(A)** rank the contribution of testosterone-axis variables to the XGBoost model. The SHAP summary plot **(B)** shows the direction and distribution of feature contributions on the log-odds scale. Red indicates higher standardized feature values and blue indicates lower standardized feature values. SHAP, Shapley additive explanations; BioT, bioavailable testosterone; FT, free testosterone; SHBG, sex hormone-binding globulin; TT, total testosterone.

SHAP analysis of the XGBoost model ranked SHBG, age, BioT%, FT%, and TT as the leading contributors to model predictions. FT and BioT showed minimal additional contribution in the final XGBoost model. The SHAP-based interpretation is shown in **Figure 9**. Because the predictors were correlated and the analysis was internal only, these SHAP results should be interpreted as exploratory and hypothesis-generating rather than confirmatory.

## Discussion

In this cross-sectional analysis of 420 male patients from an andrology outpatient clinic, we evaluated associations between testosterone-related indices and glucose-lipid traits. TT was inversely associated with glucose and TG and positively associated with HDL-C. FT and BioT showed weaker associations with glucose-lipid traits. FT% and BioT% were positively associated with TG and inversely associated with HDL-C, but these associations were attenuated or even reversed after SHBG was included in the models.

### Association of TT with glucose-lipid traits

In the present study, TT was inversely associated with glucose and TG and positively associated with HDL-C, which is consistent with most previous evidence. Farooq et al. (14) reported a significant inverse correlation between fasting glucose and serum testosterone in men with diabetes (*r* = -0.252, *P* = 0.001). Grandys et al. (15) found, using multiple linear regression in 149 men, that testosterone and free testosterone levels were significantly inversely associated with TG (*P* < 0.03). Shirakawa et al. (16) also reported a significant positive association between TT and HDL-C (*P* < 0.05).

The co-occurrence of lower TT and abnormal glucose-lipid metabolism may reflect bidirectional interactions between obesity, visceral adiposity, and endocrine-axis dysfunction (17–19). First, excess peripheral adipose tissue can increase endogenous aromatase activity, converting circulating testosterone to estradiol and suppressing the hypothalamic-pituitary-gonadal (HPG) axis through negative feedback, resulting in a secondary decline in TT (20–22). Second, visceral adipose tissue releases inflammatory mediators such as tumor necrosis factor-alpha (TNF-α) and interleukin-6 (IL-6), which may inhibit hypothalamic gonadotropin-releasing hormone (GnRH) secretion, induce systemic insulin resistance in skeletal muscle and liver, reduce skeletal-muscle glucose uptake, and promote hepatic *de novo* lipogenesis, thereby contributing to stress-related increases in glucose and TG (23–25). Because BMI, WC, fasting insulin, smoking, alcohol intake, and physical activity were not available, some of the observed associations may still reflect residual confounding, and the cross-sectional design does not allow temporal ordering.

### Associations of FT and BioT with glucose-lipid traits

Compared with TT, the free fraction of testosterone is generally considered to better represent the biologically active androgen pool (26, 27). However, in this study, calculated FT and BioT concentrations showed substantially weaker associations with glucose-lipid traits than TT. Several explanations may account for this finding.

First, the Vermeulen formula commonly used in clinical practice depends heavily on the absolute concentrations of TT, SHBG, and ALB. Routine outpatient testing is usually based on a single static blood sample and cannot fully capture the circadian pulsatility and short-term physiological fluctuation of sex hormones (28). Formula-derived error in cross-sectional data may therefore attenuate the apparent peripheral metabolic effects of calculated FT and BioT.

Second, when important confounders such as body mass index (BMI), waist circumference (WC), and fasting insulin are not fully adjusted for, calculated free testosterone may be less sensitive to early mild insulin resistance than TT, which reflects the overall androgen reserve (29, 30). These results suggest that FT and BioT should be interpreted together with TT and the broader clinical context when assessing cardiometabolic risk in men. In this outpatient cohort, TT remained the most robust testosterone-related marker for glucose-lipid abnormalities, whereas FT and BioT were less informative than TT for routine metabolic assessment.

These data suggest that FT and BioT should not be interpreted separately from TT and the broader clinical context when assessing cardiometabolic risk in men.

### Associations of FT% and BioT% with glucose-lipid traits

Notably, the derived percentage indices FT% and BioT% were positively associated with TG and inversely associated with HDL-C, a pattern opposite to that observed for TT. This finding should not be interpreted as evidence that a higher proportion of free testosterone has an adverse metabolic effect.

Higher FT% and BioT% can be driven by higher absolute TT, but they can also result from lower SHBG concentrations. SHBG is a glycoprotein mainly synthesized and secreted by the liver. Beyond its function as a carrier of sex hormones, SHBG has increasingly been recognized as a sensitive marker of hepatic metabolic dysfunction (31, 32). In obesity, non-alcoholic fatty liver disease (NAFLD), and hyperinsulinemia, hepatic *de novo* lipogenesis is activated. Excess lipid accumulation and high insulin levels can jointly suppress hepatic SHBG gene transcription and secretion, leading to reduced circulating SHBG concentrations (33, 34). Lower SHBG reduces androgen-binding capacity and can thereby increase calculated FT% and BioT%.

Previous studies have shown that low SHBG is accompanied by dyslipidemic features such as high TG and low HDL-C. Jahromi et al. (35) demonstrated in a cross-sectional study that TG was significantly inversely associated with SHBG (*P* = 0.02). Liu et al. (36) provided genetic evidence from Mendelian randomization showing a significant inverse causal association between SHBG and TG (MVMR: *β* = -0.48, *P* < 0.001). Ichikawa et al. (37) reported that low SHBG was significantly associated with high TG and elevated LDL-C, suggesting that reduced SHBG often coexists with dyslipidemia. In the present study, after SHBG was added to the multivariable linear regression models, the associations of FT% and BioT% with TG and HDL-C were markedly attenuated or reversed. This suggests that the apparent association between a higher proportion of free testosterone and adverse glucose-lipid metabolism may largely reflect SHBG-related changes in testosterone distribution. Because FT%, BioT%, TT, and SHBG are mathematically coupled, these SHBG-adjusted patterns should be interpreted as SHBG-related redistribution rather than formal mediation evidence.

### RCS, age stratification, and sensitivity analyses

RCS analysis showed robust overall monotonic associations of TT with glucose, TG, and HDL-C (*P*-overall < 0.001), with no clear U-shaped or other non-linear trajectory across the observed range (*P*-nonlinear > 0.05). This suggests a relatively stable linear relationship between TT and glucose-lipid traits within the routine exposure range of testosterone in men. Although the spline curve for TC showed slight local curvature, neither the overall nor non-linear test reached statistical significance; therefore, the present data do not support a biologically meaningful non-linear association between TT and TC.

In age-stratified analyses, interaction tests (*P* for interaction > 0.05) indicated that the association between lower TT and adverse glucose-lipid traits was generally consistent across younger men (<35 years), middle-aged men (35–44 years), and older men (≥45 years). This supports the consistency of the association direction across age strata, rather than indicating definitive generalizability. In addition, after excluding the highest 1% of extreme metabolic values and applying conventional z-score and log-rank transformations, the main TT-related effect estimates remained relatively stable.

### Machine learning-assisted validation and interpretability

The machine learning-assisted analysis provided an internal validation layer for the main statistical findings. The XGBoost model did not substantially outperform the simpler Age + TT + SHBG model, indicating that the added value of complex machine learning was limited in this dataset. However, SHAP analysis ranked SHBG and age as the two most important contributors, with BioT%, FT%, and TT providing smaller additional contributions. This pattern supports the central interpretation that SHBG-related testosterone distribution is closely linked to adverse glucose-lipid metabolic status. Therefore, the machine learning results should be interpreted as exploratory validation and explanation rather than as a mature clinical prediction tool. Because the predictors were correlated and the analysis was internal only, these SHAP results should not be interpreted as proof of causality or as a definitive clinical ranking.

### Limitations and future directions

This study has several limitations. First, information on several important confounders was unavailable, including BMI, WC, visceral fat area, fasting insulin, smoking, alcohol consumption, physical activity, and other lifestyle factors; therefore, residual confounding may have biased the observed associations in either direction. Second, because the cohort was derived from an andrology outpatient setting, these findings should be interpreted in this clinical context, and further studies in broader community-based populations are warranted to confirm their generalizability. Third, the composite metabolic outcome combined multiple abnormalities, which improved internal prediction but reduced etiologic specificity. Fourth, SHBG-adjusted analyses were mechanistically suggestive but did not constitute formal mediation analysis. Fifth, the RCS and age-interaction analyses were limited by sample size, particularly in the older age group, and therefore require validation in larger cohorts. Sixth, the machine learning-assisted analysis used internal cross-validation only, without external validation.

Future prospective longitudinal studies with larger sample sizes should incorporate insulin measurements, lifestyle variables, and external validation cohorts. In addition, direct comparisons between mass spectrometry-based measurement of free testosterone and mathematical formula-derived estimates are needed to determine their consistency in metabolic prediction and to provide stronger evidence for the association between testosterone-related indices and glucose-lipid metabolism.

## Conclusion

This study showed that low TT in male andrology outpatients was robustly and linearly associated with higher glucose, higher TG, and lower HDL-C, with generally consistent associations across age strata. Compared with FT and BioT, TT appeared to be more sensitive for reflecting abnormalities in glucose-lipid metabolism. In clinical interpretation of male sex-hormone reports, high FT% or BioT% should not be used alone to infer a favorable androgen state; TT and SHBG concentrations should be considered together. The exploratory machine learning-assisted analysis further supported the importance of SHBG-related features in identifying adverse glucose-lipid metabolic status, although the predictive performance remained moderate and requires external validation. These findings should be interpreted as cross-sectional associations in a specialty outpatient cohort rather than as evidence of causality or broad population generalizability.

## Data Availability

The original contributions presented in the study are included in the article/supplementary material. Further inquiries can be directed to the corresponding author.
